# Rethinking the beginning of the ‘nuclear age’ through telling feminist nuclear stories

**DOI:** 10.1007/s42597-022-00082-8

**Published:** 2022-12-29

**Authors:** Laura Considine

**Affiliations:** University of Leeds, Leeds, UK

**Keywords:** Nuclear weapons, Feminism, Everyday, Nuclear history, Narrative, Atomwaffen, Feminismus, Alltag, Nukleare Geschichtsschreibung, Narrativ

## Abstract

Bringing a feminist perspective to the global politics of nuclear weapons not only allows us to expand who and what counts as worthy of study in nuclear politics, but also contests core ideas and narratives that have shaped the literature to date. This paper asks how telling feminist nuclear stories rooted in an understanding of the everyday impacts of nuclear weapons challenges the traditional nuclear history. The standard history of ‘the bomb’ focuses on the military-industrial development of the Manhattan Project in the context of World War II, focusing on the figure of the elite male scientist as embodying the unique moral dilemmas of a new ‘nuclear age’. Feminist stories of the development of the first atomic weapons can instead illuminate the marginalised bodies and expansive, everyday harms of the development of nuclear weapons technology, altering both the timeline and the space of nuclear politics.

## Abstract (de)

Eine Betrachtung der globalen Atomwaffenpolitik aus feministischem Blickwinkel ermöglicht nicht nur eine Erweiterung des Personenkreises und der Sachverhalte, die im Bereich der Atompolitik als untersuchungswürdig gelten, sondern zieht auch zentrale Vorstellungen und Erzählungen in Zweifel, welche die Literatur bis heute prägen. Die vorliegende Arbeit wirft die Frage auf, wie feministische Schilderungen zum Thema Kernwaffen, verwurzelt in einem Verständnis der alltäglichen Auswirkungen von Kernwaffen, die traditionelle nukleare Geschichtsschreibung infrage stellen. Die geschichtliche Standarderzählung von „der Bombe“ konzentriert sich auf die militärisch-industrielle Entwicklung des Manhattan-Projekts im Rahmen des Zweiten Weltkriegs und legt den Fokus dabei auf die Gestalt des männlichen Spitzenwissenschaftlers als Verkörperung des singulären moralischen Dilemmas eines neuen „nuklearen Zeitalters“. Feministische Schilderungen der Entwicklung erster Kernwaffen können stattdessen die marginalisierten Gruppen und weitreichenden, alltäglichen Schäden durch die Entwicklung von Kernwaffentechnik beleuchten, unter Modifikation von Zeitleiste und Raum der Atompolitik.

## Introduction

The pilot episode of the United States television programme *Manhattan, *about the invention of nuclear weapons, opens in the New Mexico desert. Text appears on the screen: ‘July 2 1943. 61 countries at war. More than 40 million casualties.’ The figure of a white male scientist, Dr Frank Winter, becomes partly visible through dust-filled desert air. It is night and he is standing in the dark, buffeted by sandy wind, lit only by his car headlights. Back in the car he scribbles on pieces of paper while smoking a cigarette, clearly working on a problem and finding no solution. Outside the car again he swings a golf club and hits a ball into the gaping dark. He gazes after his shot into the night, takes another golf ball from his pocket, stares intently at it and a half smile flits across his face; ‘son of a bitch’ he mutters before rushing to his car and speeding away. He has clearly been struck by a deeply important insight. The following day we learn the nature of this flash of inspiration back at the Los Alamos weapons laboratory: alone in the desert night he intuited from the compression of the golf ball when struck by the club the potential value of compression for the fissile material at the core of an atomic bomb.

This opening scene of a television drama about Los Alamos scientists acts as a perfect distillation of the dominant popular account of the development of nuclear weapons in the Manhattan Project; a story that has been told countless times across multiple media, from graphic novel to opera.^1^ Almost eight decades after the first test of an atomic bomb, this particular retelling of the nuclear origin story clearly repeats longstanding tropes of what I have called the ‘nuclear origin myth’ (Considine 2021a, see also Norris 1997). The nuclear origin myth refers to a dominant western, and mostly American, retelling of the history of the Manhattan Project in popular culture and scholarship, produced by and about a group of elite insiders from positions of relative power.^2^ The myth tells a specific story about the nuclear past that has become dominant in popular culture and much academic work, becoming the ‘common sense’ understanding of the story of the development of the first US nuclear weapons. This story is one of progress in which nuclear weapons are ‘symbolic objects of Western modernity’ (Considine 2021a, p. 2). It is also a story of inevitability in which the power of splitting the atom, once known, will certainly and inescapably be used—the only question is by whom. Finally, it is also the story of men and masculinity, in particular the tragic male figures of the Manhattan Project scientists whose brilliance is matched in size only by the scale of the moral dilemmas they faced in developing atomic weapons. The lone figure of Dr Frank Winter standing in the grimy dark, lighting the way towards the bomb through a flash of individual brilliance in the opening scenes of Manhattan provides a visual encapsulation of the nuclear origin myth’s concentration on the figure of the male scientist as the tragic protagonist of the nuclear origin story. It also visually captures the prevailing narrative of the development of these weapons as one of revelation: uncovering the secret of a universal force that is just waiting to be unleashed.

## The impacts of the nuclear origin myth

The fixation on the scientists at Los Alamos and a few other laboratories shows little sign of waning as acclaimed Hollywood film writer and director Christopher Nolan is currently directing a new biographical film about Los Alamos Director J. Robert Oppenheimer, based on the biography *American Prometheus* written by Kai Bird and Martin J. Sherwin (2005). Oppenheimer more than any other figure has come to personify the image of the nuclear scientist and the dilemmas of the nuclear age. But the Manhattan Project was more than a few famous scientists and the focus on Oppenheimer and a select group of elite men obscures the wide range of other stories to be told about the development of nuclear weapons. At its peak over 130,000 people were employed across thirty sites—the Manhattan Project was a sprawling military industrial behemoth. At Los Alamos, many of these employees were not white scientists but native and Pueblo workers, previously forced off their land and now employed at the site in roles such as maids, cleaners and construction workers (Gusterson 1996). Although the nuclear world created by the Manhattan Project story was male and masculinist, the workers at assembly lines handling uranium products were often women and generally working-class women. These women laboured with no idea of what they were working towards, and no knowledge of any health dangers connected to their labour. The story of nuclear origins is not even limited to the workers and sites of the Manhattan Project but includes the places from where nuclear materials were extracted. For example, the majority of the uranium used in the first atomic weapons came from Shinkolobwe in the (then called) Belgian Congo where local workers were forced to mine in unsafe conditions (Hecht 2012). Who is telling these stories? Feminist scholarship is well-placed to do so, but while nuclear weapons have always laboured under ‘the ubiquitous weight of gender’ (Cohn 1987, 688), there has been a lack of sustained feminist work on the issue in the post-Cold War era.^3^

How nuclear history is narrated matters, not just in terms of providing a full and accurate representation of a nuclear past, but because of the socio-political function these historical stories have in the nuclear present. Dominant narratives such as that of the nuclear origin myth give a particular social meaning to nuclear weapons (Considine 2021b). The origin myth story signals to the listener that the moral dilemmas and perspectives of western male scientists are *the* dilemmas and perspectives from which to understand nuclear weapons. It obscures the labour of hundreds of thousands of people, the extractive and often exploitative process of mining, processing, and enriching uranium and the lasting impacts of this process on communities and places far away from Los Alamos.

## Challenging the fetishization of ‘the bomb’

It is important therefore to interrogate the dominant narrative of nuclear origin and its implications. The fixation of the figure of the male scientist as embodying the story of the Manhattan Project and the political and moral dilemmas accompanying the nuclear weapons complex has been noted by authors including Gabrielle Hecht. She notes how scholars (and I would add popular culture) have ‘fetishized “the bomb” and its builders. Witness the obsession with the historical minutiae of “the decision to drop the bomb,” the endless stream of biographies of Manhattan project scientists, and the insistence on the uniqueness of moral dilemmas posed by atomic activities’ (Hecht 2012, p. 13). This treatment of the nuclear as a unique and ontologically separated realm embodied by the male scientist produces and reproduces the idea of nuclear exceptionalism (Hecht 2012, p. 6), that nuclear things are self-evidently out of the ordinary and the everyday. Instead, Hecht shows, through detailed study of a history of the occupational hazards of uranium mining in Africa and Africa’s place in the nuclear world, that nuclearity is ‘a contested technopolitical category’ (Hecht 2012, p. 14) and one that is gendered.

Gabriele Schwab describes the Manhattan Project as ‘an almost orgiastic culmination of male fantasies of conquest’ in which the ‘scientists who worked in Los Alamos to prepare the testing and eventual use of the first nuclear weapons are carried away by a morbid fascination with the nuclear sublime that culminated in a veritable sacrilization of the Bomb’ (Schwab 2020, p. 120). Like Hecht, Schwab connects the fantasy of ‘the bomb’ as an exceptional and sublime object to its creation through and by masculinity. The story of the nuclear origin reproduces a fascination with nuclear sublime and a ‘sacralisation of the bomb’ with associated redemptive fantasies based on a delusion of masculine control over nature. Indeed, Schwab states that the gendered nuclear imaginary of nuclear control and fascination with the nuclear sublime is intimately connected with a fantasy of male birth and of the atomic bomb as a ‘bachelor machine’ (Carrouges in Schwab 2020, p. 119). The bachelor machine symbolises fantasies of male generation of a new species, revealing ‘a tacit politics of reproduction’ and ‘womb envy’ (Schwab 2020, p. 119). This new species is male, as ‘emblematized by the cold cynicism of naming the first atomic bomb dropped over Hiroshima “Little Boy”’ (Schwab 2020, p. 119). The use of metaphors and images of male birth suffuse the discourse, for example, George L. Harrison, advisor to Secretary of War Henry L. Stimson famously reported the test of the first atomic bomb with the message ‘Doctor has just returned most enthusiastic and confident that the little boy is as husky as his big brother. The light in his eyes discernible from here to high hold and I could have heard his screams from here to my farm’ (Harrison 1945). With the new male birth of the atomic bomb, the world itself is reborn into the ‘nuclear era’. Even if one doesn’t adopt the psychoanalytic lens taken by Schwab, the American story of nuclear ‘birth’ is a discursively male one.^4^

Hecht and Schwab’s interpretation of the fetishization of masculinity in the Manhattan Project narrative fits within a wider narrative of nuclear strategy and policy-making as a historically male-dominated and masculinised space. In this space a nuclear ‘brotherhood’ reproduces technostrategic discourses of virility, dominance, and rationality (Cohn 1987; Easley 1983). In contrast, women’s voices have been most amplified in anti-nuclear protest, often with a focus on motherhood or the essentialised pacifism of women.^5^ Feminist work shows how such gendered spaces and practices have shaped nuclear political discourse through dichotomies of masculine-feminine and the ‘symbolic gendered dimensions of nuclear weapons’ (Cohn et al. 2005), for example associating weapons capability with virility, disarmament with feminised weakness and ‘serious’ research on nuclear weapons with a militarised or scientific masculinity. This can also be seen in gendered and orientalist ideas about the ‘responsible stewardship’ of nuclear weapons, in which certain states are deemed rational, advanced and thus capable of the responsibility of nuclear weapons possession and its concomitant protector status (Duncanson and Eschle 2008). Such gendered divisions are also racialised, in which the western white male is separated from and superior to the non-western ‘other’ (Gusterson 1999). The dominant narrative of the development of nuclear weapons in the Manhattan Project reproduces such understandings. The male scientists of the Manhattan Project have been elevated as subjects of unique fascination and gravity, the retelling of the story of ‘the bomb’ expressed in reverent tones and through images of birth, eternity and godly power, the moral dilemmas of the scientists represent the ‘good’ rational steward of atomic power, one who understands and accepts the grave responsibility of managing nuclear weaponry. All of which perpetuates the gendered dynamics of nuclear politics identified by feminist work.

Feminist thinking therefore, provides ways through which to interrogate the contents and impacts of popular nuclear stories and in particular of the stories of the nuclear beginning. However, there is a conspicuous lack of sustained gender-focused and feminist academic research on the topic since pioneering and influential works of the 1980s and early 1990s. Carol Cohn’s 1987 article ‘Sex and Death in the Rational World of Defense Intellectuals’ is rightly considered a foundational and trailblazing text on the gendered dimensions of nuclear weapons and still has much to say today, yet it is also notable that an article written thirty five years ago during the Cold War remains the ‘go-to’ academic feminist work on nuclear weapons. This scholarly gap goes both ways in that those studying nuclear weapons as their primary research topic have generally neglected questions of gender and rarely adopted feminist approaches. In addition, post-Cold War feminist International Relations (IR), as well as other ‘critical’ strands of IR and security studies, has tended to overlook nuclear weapons as a site of scholarship. Work on nuclear weapons has not benefited from the developed literature and critical insights on other aspects of feminism and IR that would provide theoretical and methodological tools through which to analyse how the politics and history of nuclear weapons are understood and practiced. Incorporating more feminist IR literatures and practices in the study of nuclear weapons can provide the conceptual apparatus for any project that seeks to reimagine the knowledge structures and institutions of nuclear politics that reproduce gendered and racialised violence through telling feminist stories about the nuclear past. There is great potential for research investigating how the nuclear origin myth as a dominant retelling of the nuclear past maintains the contours of nuclear policymaking today.

## Telling feminist stories of nuclear beginning

So how can we begin to tell more feminist stories about the nuclear beginning? Below are a few reflections, they are by no means definitive or even fully finished but are simply some paths I see forward for the future of the nuclear past. One step is to reconsider whose story gets told, remembered, and commemorated and to ask Cynthia Enloe’s question of ‘where are the women?’ (1990). While the agonised decision-making of the elite men making the first atomic weapons has been lionised in popular culture, there is limited discussion of the fact that most workers, including a vast majority of the women working on the Manhattan Project doing assembly line tasks, had no ability to make such judgements either about their willingness to partake in such a project or on the impacts of such work on their own health (Schwab 2020). While there is some research on the role of women in the Manhattan Project (Kiernan 2013; Rall 2006; Howes and Herzenberg 1999), more could be done.

Simply adding women’s stories to the nuclear origin myth is, however, not adequate to challenge its socio-political implications and does not necessarily mean one is telling a feminist story. Rather, given that the Manhattan Project was embedded in colonial, racialised structures and practices, a feminist narrative may not just be one that tells the story of women but rather one that has an orientation ‘towards marginalised bodies and sites that start with a feminist politics that take women and gender seriously; however, where this departure point takes any given feminist research and politics are infinite.’ (choi 2021, p. 63 see also Richter-Montpetit 2016). There is great potential for research that studies the marginalised bodies and sites of the Manhattan Project and their gendered socio-political dynamics and contexts. For example, at the Hanford facility in Washington State where the plutonium in the Fat Man bomb dropped over Nagasaki was produced, Du Pont Corporation management paid African American and Mexican Americans less than white workers, setting up a ‘coloured barracks for African Americans and forcing its Mexican American employees to ride a bus 60 miles to the plant to work and segregating the site’, in doing so it ‘introduced racial segregation into eastern Washington’ (Brown 2013, p. 27). This story is just one example of marginalised bodies and spaces in the gendered fantasy of the Manhattan Project that warrants further study.

A second step towards telling feminist stories could be to rethink the way in which the nuclear origin myth locates the atomic weapon along a historical continuum of violence and progress: as the outcome of a long history of humankind’s war-making tendencies as well as the outcome of the development of enlightenment thinking and scientific discovery. It is possible to narrate the development of the atomic bomb as located along a different (though intimately related) timeline, one of a history of extraction and colonialism (Biswas 2014; Teaiwa 1994). Feminist work has asked us to reconsider what a continuum of violence means in relation to the Manhattan Project and how violence occurs not just in time but that ‘at linked points along other continuua too—of space and place (bedroom to battlefield), of scale (fist to bomb) and of type or kind (cultural and institutional, overt and direct)’ (Cockburn 2004, p. 357). By centring the everyday, the gendered and marginalised bodies and sites it is possible to also locate the atomic bomb along violent continuua of place, scale and type. One can see how, for example, the racialised violence at Hanford is inextricable from the violence of the nuclear explosion at Nagasaki made with Hanford plutonium. Or how the forced expulsion of Pueblo and native New Mexicans from the land taken for use on Los Alamos and the return of these communities to the site as maids and janitors are at once a specifically located violence and at the same time connected with the tests of US thermonuclear weapons in the Marshall Islands in Pacific, whose peoples were also displaced in service of ‘the bomb’.

Indeed, the legacy of these displacements continues to shape lives in multiple unexpected ways today. For example, during the first months of the COVID-19 pandemic, the Marshallese community living in northern Arkansas (the largest in the continental US with 20,000 residents) suffered 38% of COVID-19 fatalities, despite making up less than 4% of the population, because of their working and healthcare situations (Sy 2022). Many Marshallese in Arkansas worked in cramped conditions in poultry processing plants, which remained open while other workplaces closed. Their special immigration status, which is linked to their displacement by US nuclear tests means they had limited access to healthcare, with no access to US social medicine programme Medicaid until December 2020. The healthcare outcomes of displaced Marshallese almost seven decades after the US began nuclear testing in the Pacific can be located on a continuum of violence alongside the Manhattan Project and the first nuclear test. Such a perspective challenges the idea that the origin story of the atomic weapon is one of abstract ideals and agonises about nuclear salvation or apocalypse, embodied by elite male scientists. It instead shows the multiple webs of effect, violence and harm of the development of nuclear weapons, which is still missing from too much of the popular and academic nuclear storytelling.

## Footnotes

1 See, for example, Rhodes (1986), Bird and Sherwin (2005), Pais and Crease (2007), Schweber (2000), Herken (2003), for a fuller examination see Considine (2021a).

2 For example, Manhattan Project scientists, journalists with access, official historians. For a study of the ‘Americanisation’ of the Manhattan Project see Laucht (2009).

3 Notable exceptions include Acheson (2018, 2021); Duncanson and Eschle (2008); Eschle (2013, 2017); Das (2007, 2010); Runyan Sisson (2018). Scholarship is beginning to address this gap, for example the recent special section on ‘Feminist interrogations of global nuclear politics’ in International Affairs (July 2022).

4 Recent work by Hebatalla Taha (2022) on visualisations of the bomb in Egypt shows an alternative to the dominant discourse in which the bomb is feminised, challenging feminist scholars to further examine the gendered construction of nuclear discourses outside the west.

5 For work engaging with and complicating this representation of women’s protests see Eschle (2017).
